# Biventricular differences in β-adrenergic receptor signaling following burn injury

**DOI:** 10.1371/journal.pone.0189527

**Published:** 2017-12-12

**Authors:** Ashley N. Guillory, Robert P. Clayton, Anesh Prasai, Amina El Ayadi, David N. Herndon, Celeste C. Finnerty

**Affiliations:** 1 Department of Surgery, University of Texas Medical Branch, Galveston, Texas, United States of America; 2 Shriners Hospitals for Children^®^—Galveston, Galveston, Texas, United States of America; 3 Institute for Translational Sciences, University of Texas Medical Branch, Galveston, Texas, United States of America; University of Oslo, NORWAY

## Abstract

Burn injury detrimentally affects the myocardium, primarily due to over-activation of β-adrenergic receptors (β-AR). Autopsy reports from our institution reveal that patients often suffer from right ventricle (RV) failure. Since burn injury affects β-AR signaling in the left ventricle (LV), we proposed that β-AR signaling may also be altered in the RV. A rodent model with a scald burn of 60% of the total body surface area was used to test this hypothesis. Ventricles were isolated 7 days post-burn. We examined the expression of β-ARs via Western blotting and the mRNA expression of downstream signaling proteins via qRT-PCR. Cyclic adenosine monophosphate (cAMP) production and protein kinase A (PKA) activity were measured in membrane and cytosolic fractions, respectively, using enzyme immunoassay kits. β_1_-AR protein expression was significantly increased in the RV following burn injury compared to non-burned RV but not in the LV (p = 0.0022). In contrast, β_2_-AR expression was unaltered among the groups while G_αi_ expression was significantly higher in the LV post-burn (p = 0.023). B-arrestin-1 and G-protein coupled receptor kinase-2 mRNA expression were significantly increased in the left ventricle post-burn (p = 0.001, p<0.0001, respectively). cAMP production and PKA activity were significantly lower in the LV post-burn (p = 0.0063, 0.0042, respectively). These data indicate that burn injury affects the β-AR signaling pathway in the RV independently of the LV. Additionally, non-canonical β-AR signaling may be activated in the RV as cAMP production and PKA activity were unchanged despite changes in β_1_-AR protein expression.

## Introduction

Cardiovascular dysfunction after severe burn injury has been demonstrated clinically in patients as well as in rodent and mammalian burn models. [1, 2] However, the molecular mechanisms underlying this cardiac dysfunction are not clearly defined. We have shown in severely burned pediatric patients that the release of the catecholamines epinephrine and norepinephrine are significantly enhanced after injury and this increase in release is associated with perturbations of heart rate and cardiac work. [2, 3] Additionally, autopsy reports from our institution indicate that many pediatric patients present with right heart failure. [4]

Circulating catecholamines activate myocardial adrenergic receptors to modulate cardiac function. [5] Increased catecholamine levels contribute to the development and progression of cardiac dysfunction in several disease states, including heart failure. [6, 7] While there are several types of adrenergic receptors in the heart, altered catecholamine levels’ effect on β_1_- and β_2_-adrenergic receptor (β_1_-AR, β_2_-AR) signaling has been well studied in various non-burned states. Overstimulation of β-ARs by catecholamines results in dysregulation of the associated signaling pathways. Among other things, there can be desensitization of the receptors, altered protein phosphorylation, and activation of alternate signaling pathways. [8]

Both right and left ventricles predominately express β-ARs, but α-adrenergic receptors also modulate cardiac function. It has also been hypothesized that the right ventricle is more dependent upon α-adrenergic receptor activity than the left ventricle. The right and left ventricles respond to catecholamine administration in vastly different manners. Irlbeck et al., reported that norepinephrine infusion resulted in altered right ventricular hemodynamics but left ventricular hypertrophy. [9] The two ventricles develop from different cellular lineages, which may partially explain why each ventricle responds differently to stimuli. Based on the clinical and basic science evidence, we hypothesized that burn injury would affect β-AR signaling differentially in the left and right ventricles.

## Methods

### Animal model of burn injury

The Institutional Animal Care and Use Committee of University of Texas Medical Branch-Galveston in accordance with the National Institutes of Health *Guide for the Care and use of Laboratory Animals* approved this study’s protocol (Protocol#:0506032). A full-thickness scald was induced in male Sprague-Dawley rats as previously described. [10, 11] Briefly, rats (n = 9–12 per group) were acclimated for 1 week in the animal care facility prior to experimentation. Animal subjects were housed under standard laboratory conditions with water and food available ad libitum. Prior to the burn, all animals (control and burn) received 0.05mg/kg Buprenorphine. 1–3% isoflurane in air was used to induce general anesthesia. Sufficient depth of anesthesia was determined using the toe pinch method with nonserrated forceps. A scald burn covering 60% of the total body surface area was administered to the shaved dorsal and lateral surfaces. Resuscitation was achieved with administration of 40ml/kg Lactated Ringers solution. Animals were recovered with oxygen under observation and placed in wire bottom cages for the duration of the experiment. Animals were housed individually to prevent barbering and reduce infection. Laboratory personnel and veterinary staff rounded on animals several times daily. If a sufficient score on the pain scale was observed, 0.05 mg/kg Buprenorphine was administered every 8–12 hours until pain was no longer evident. At the conclusion of the study, animals were euthanized via decapitation without anesthesia.

### Experimental design

The groups for this experiment were control and burn. Control animals received analgesia, anesthesia, and were shaved but did not undergo the burn procedure or resuscitation. We chose 7 days post-burn as the study period because of previously published data showing that there were alterations in β-ARs, cyclic adenosine monophosphate (cAMP) production, and protein kinase A (PKA) activity 1 week after burn injury. [12]

### Western blotting

β_1_-AR, β_2_-AR, β_3_-AR (Santa Cruz Biotechnology, Santa Cruz, CA), G_αs_, G_αi_, and GAPDH (Cell Signaling Technology, Danvers, MA) protein expression was determined via Western Blot analysis. Thirty micrograms of left and right ventricular homogenates were resolved on SDS-PAGE gradient gels (4–15%, Tris-Glycine) and transferred to polyvinylidine difluoride membranes. Primary antibody incubation occurred at 4°C for a minimum of 16 hours. Blots were then washed with 0.1% TBST, followed by incubation with anti-HRP secondary antibodies (Cell Signaling) for a minimum of 1 hour at room temperature. Band intensities were visualized using chemiluminescence (SuperSignal West Pico Chemiluminescent Substrate, Thermo Scientific, Rockford, IL, USA) and quantified using NIH ImageJ Data Acquisition Software (National Institutes of Health, Bethesda, MD, USA).

### Enzyme immunoassay kits

cAMP production and PKA activity were measured using commerically available kits from Enzo LifeSciences, Ann Arbor, MI. Aliquots of 30ug of ventricular membrane fraction was used to determine cAMP production as previously described. [13] Experiments were performed in duplicate and the amount of cAMP produced (pmols) was normalized to the protein concentration of each sample. PKA activity was determined in 20ugs of ventricular cytosolic fractions via an enzyme immunoassay kit (Enzo LifeSciences, Ann Arbor, MI). Nonspeicifc kinase activity was determined by utilizing10μM peptide inhibitor of PKA (PKI). Experiments were performed in triplicate and the relative kinase activity was normalized to the protein concentration of each sample.

### Quantitative-Real Time-PCR (qRT-PCR)

Left and right ventricular tissue was homogenized and RNA extracted using Ambion TRIzol Reagent (Life Technologies/ThermoFisher Scientific, Waltham, MA) and the RNeasy Mini Kit (Qiagen, Frederick, MD). cDNA was generated using 250ng of purified RNA and the iScript cDNA Synthesis Kit (Bio-Rad Laboratory, Hercules, CA). cDNA products were amplified in triplicate using 10ul iTaq Universal SYBR Green Supermix and 400nm primer mix to a total volume of 20ul (Table 1). Samples were normalized to ribosomal protein L13a (Rpl13a). Data are expressed as fold change compared to controls using the comparative C_T_ method. [14] Two validations were performed to ensure that there was no genomic DNA amplification. First, the melting curve was performed and analyzed to ensure that only peak was observed per gene of interest. Secondly, following the PCR amplification, gel electrophoresis was performed and a single band was observed in the gel.

**Table 1:** Primers for qRT-PCR.

β-arrestin-1 forward primer	5’- ATG CCT ACC CCT TCA CCT TT-3’
β-arrestin-1 reverse primer	5’- CCT CTC AGG GGC ATA TTG AA-3’
Vascular endothelial growth factor-a forward primer	5’- GCA ATG ATG AAG CCC TGG AG-3’
Vascular endothelial growth factor-a reverse primer	5’- GAC CCT TTC CCT TTC CTC GA-3’
Vascular endothelial growth factor-b forward primer	5’- GCA AGA ATA AAG AGG GGC CG -3’
Vascular endothelial growth factor-b reverse primer	5’- ACC CTG AAC CTT TGA GTG CT-3’
Insulin-like growth factor-1 forward primer	5’- GCT CTT CAG TTC GTG TGT GG-3’
Insulin-like growth factor-1 reverse primer	5’- CAA CAC TCA TCC ACA ATG CC-3’
G protein coupled receptor kinase 2 forward primer	5’- ATG CAT GGC TAC ATG TCC AA-3’
G protein coupled receptor kinase 2 reverse primer	5’- CCA CCT CGG ATC TTA AGC AG-3’
Ribosomal protein L13a forward primer	5’- GGA TCC CTC CAC CCT ATG ACA-3’
Ribosomal protein L13a reverse primer	5’-CTG GTA CTT CCA CCC GAC CTC-3’

### Statistical analysis

Data were analyzed with one-way ANOVA followed by Tukey-Kramer’s *post hoc test* (GraphPad Prism 5.0). Data are expressed as means ± standard error of the mean. A p-value <0.05 was considered significant.

## Results

### β-AR and G-protein expression is altered differentially in the ventricles after burn injury

**Fig 1:**
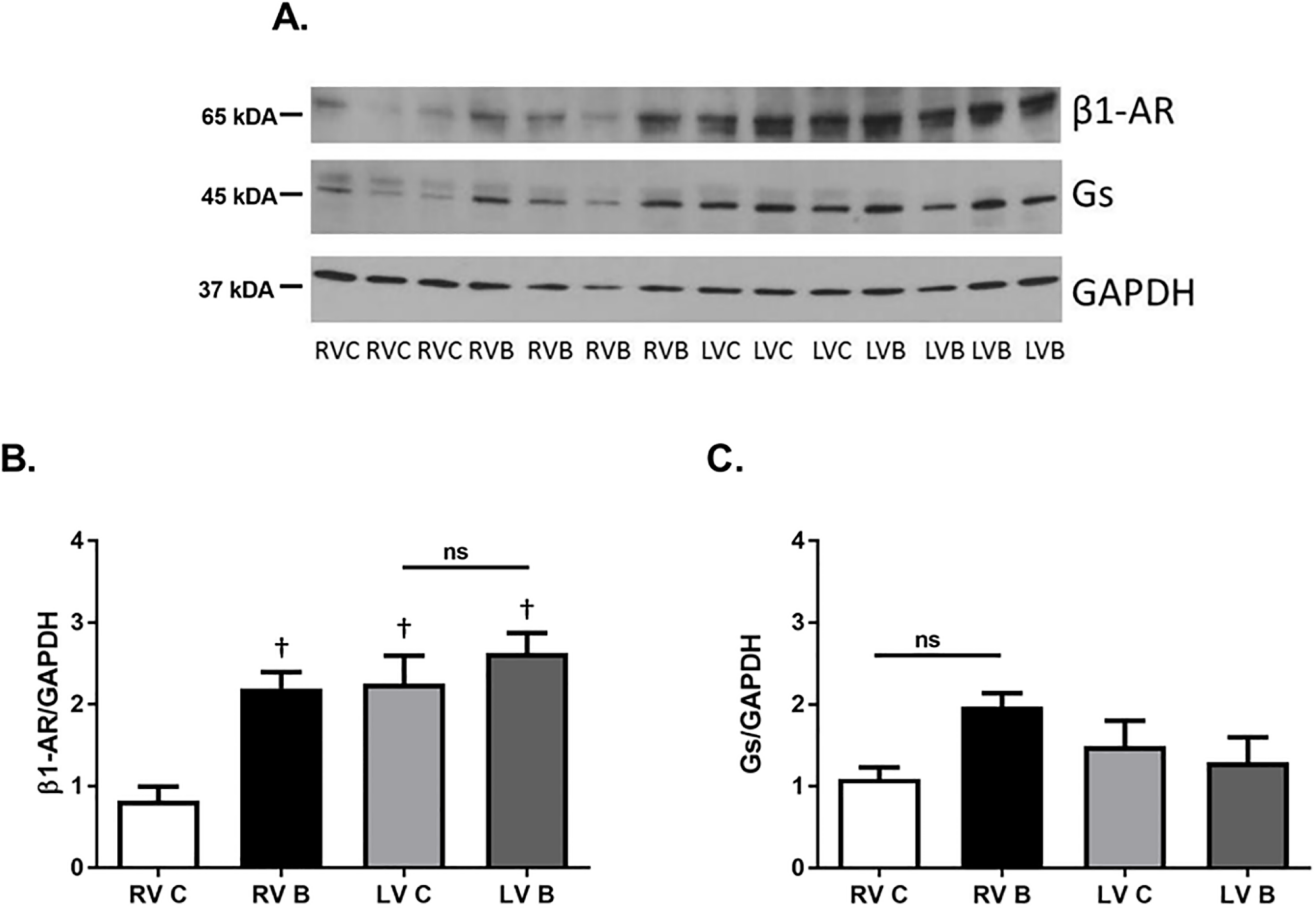
β_1_-AR and G_s_ protein expression post-burn. Representative Western blots of β_1_-AR (A, B) and G_s_ (A, C) protein expression in right and left ventricles seven days post-burn. Controls were nonburned animals. The *bar* graphs show the ratio of protein to GAPDH. Data are expressed as the mean ± SEM. Statistical analysis was performed using a one-way ANOVA. n = 9–12; p<0.00001 for β_1_-AR; RV C: right ventricle control; RV B: right ventricle burned; LV C: left ventricle control; LV B: left ventricle burned; †, p<0.05 vs RV C.

**Fig 2:**
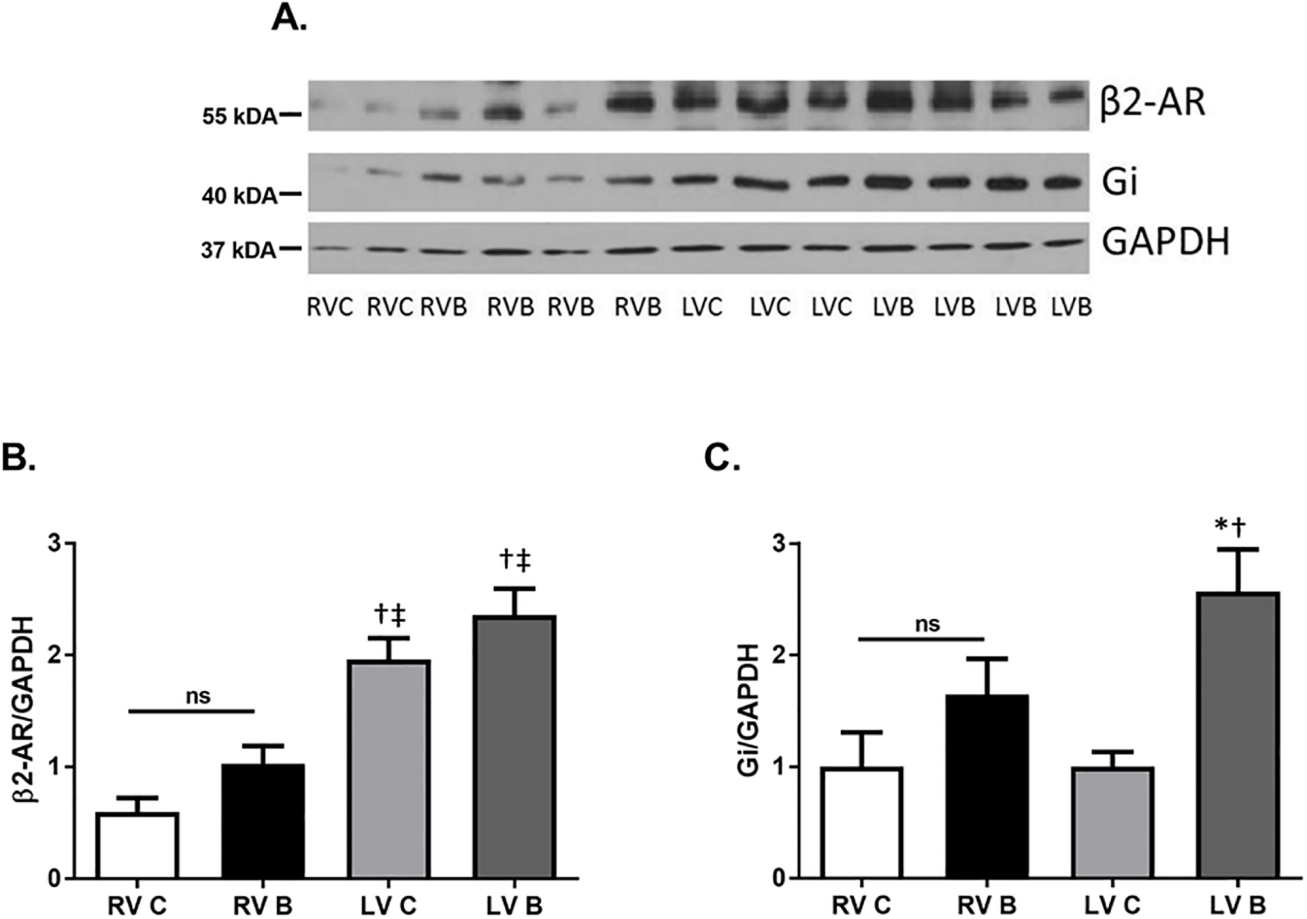
β_2_-AR and G_i_ protein expression post-burn. Representative Western blots of β_2_-AR (A, B) and G_i_ (A, C) protein expression in right and left ventricles seven days post-burn. Controls were nonburned animals. The *bar* graphs show the ratio of protein to GAPDH. Data are expressed as the mean ± SEM. Statistical analysis was performed using a one-way ANOVA. n = 9–12; p<0.0001 for β_2_-AR; p = 0.011 for G_i_; RV C: right ventricle control; RV B: right ventricle burned; LV C: left ventricle control; LV B: left ventricle burned; β-AR, beta adrenergic receptor; †, p<0.05 vs RV C; ‡, p<0.05 vs RV B.

**Fig 3:**
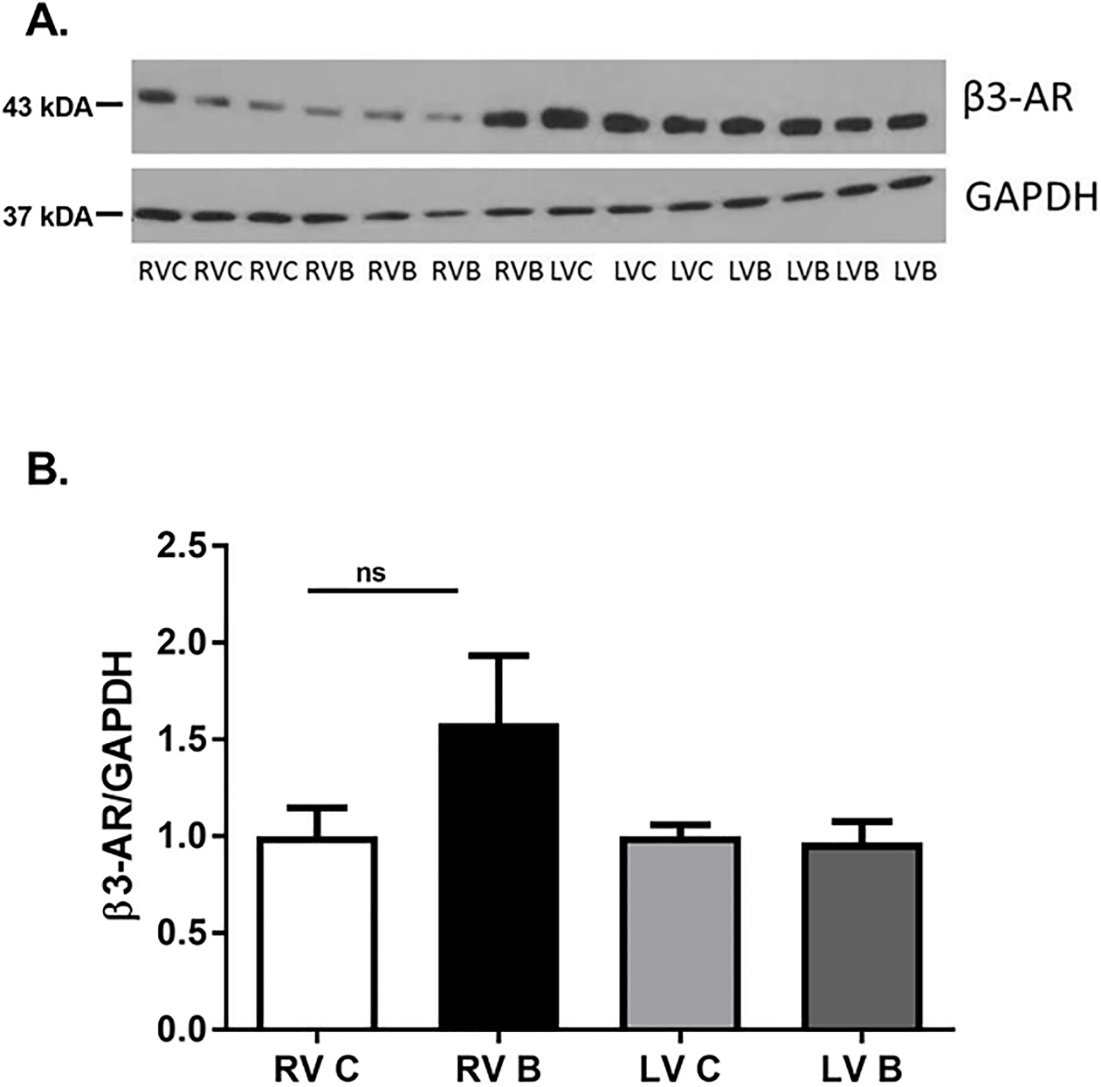
β_3_-AR protein expression post-burn. Representative Western blots of β3-AR protein expression in right and left ventricles seven days post-burn. Controls were nonburned animals. The *bar* graphs show the ratio of protein to GAPDH. Data are expressed as the mean ± SEM. Statistical analysis was performed using a one-way ANOVA. n = 6; RV C: right ventricle control; RV B: right ventricle burned; LV C: left ventricle control; LV B: left ventricle burned; β-AR; beta adrenergic receptor.

Numerous studies have revealed that severe burn injury dramatically increases the release of endogenous catecholamines epinephrine and norepinephrine. [3] In heart failure, another disease associated with increased catecholamine release, the expression levels of cardiac β-ARs are drastically different than that seen under normal conditions. [8] We first examined whether burn injury altered expression of the β-AR subtypes as well as the G-proteins most highly associated with their function. Prior to burn, left ventricular β_1_-AR protein expression was significantly higher than expression in the right ventricle (Fig 1A and 1B; p = 0.002). However, β_1_-AR protein expression was significantly increased (168%) in the right ventricle after burn injury but not in the left ventricle (p = 0.002). While there was a trend for stimulatory G-protein (G_αs_) expression to be increased in the right ventricle after burn, this was not significant (Fig 1A and 1C). β_2_-AR protein expression was not altered in either ventricle after burn injury but there was significantly higher β_2_-AR expression in the left ventricle compared to the right (Fig 2A and 2B; p = 0.006). In contrast, the inhibitory G-protein (G_αi_) was expressed higher in the left ventricles of burned animals (Fig 2A and 2C; p<0.0001). Burn injury did not alter β_3_-AR expression in either ventricle (Fig 3A and 3B).

### Increased left ventricular gene expression of proteins involved in β-AR desensitization following burn injury

**Fig 4:**
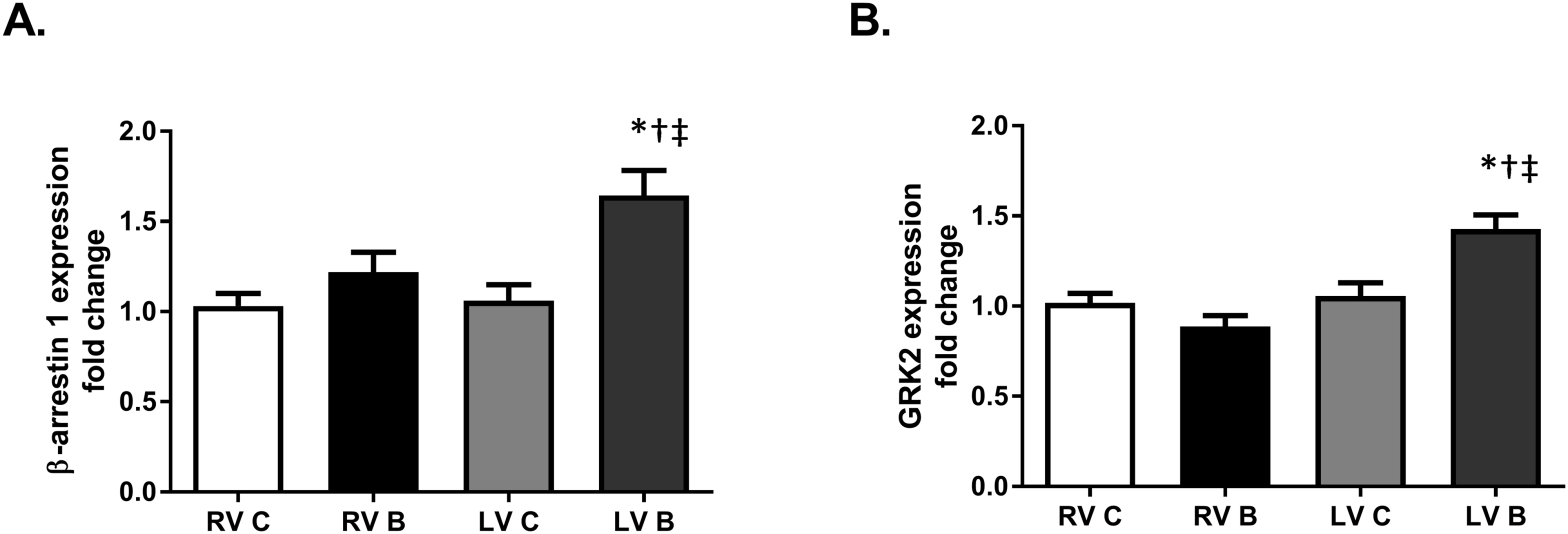
Gene expression of β-AR desensitization proteins post-burn. (A) β-arrestin 1 mRNA expression from right and left ventricles and (B) GRK2 mRNA gene from right and left ventricles at seven days post-burn. Data are expressed as the mean ±SEM. Statistical analysis was performed using a one-way ANOVA. n = 7–9; p = 0.001, <0.0001 respectively; RV C: right ventricle control; RV B: right ventricle burned; LV C: left ventricle control; LV B: left ventricle burned; GRK2, G-protein coupled receptor kinase 2; *, p<0.05 vs LV C; †, p<0.05 vs RV C; ‡, p<0.05 vs RV B.

G protein coupled receptor kinase 2 (GRK2) and β-arrestin-1 are two proteins involved in β-AR desensitization to terminate signaling through the receptors in response to increased and/or prolonged stimulation by circulating catecholamines or administration of β-AR agonists. [15, 16] Similar to what has been reported in heart failure, we observed significantly increased β-arrestin-1 and GRK2 gene expression in the LV after burn injury (Fig 4A and 4B; p = 0.001; <0.0001). Neither GRK2 nor β-arrestin-1 gene expression was altered after burn injury in the RV. Previous studies in both human and animal subjects have shown that burn injury produces a systematic and prolonged increase in catecholamine levels [11, 17]. While we did not measure catecholamines directly in this study, the alterations in GRK2 and β-arrestin-1 mRNA are indicative of desensitization of left ventricular β-ARs due to overstimulation [18, 19]. This suspected desensitization may be the mechanism by which cAMP production and PKA activity are decreased despite β-AR expression remaining constant.

### Burn injury reduces left ventricular cAMP production and PKA activity

**Fig 5:**
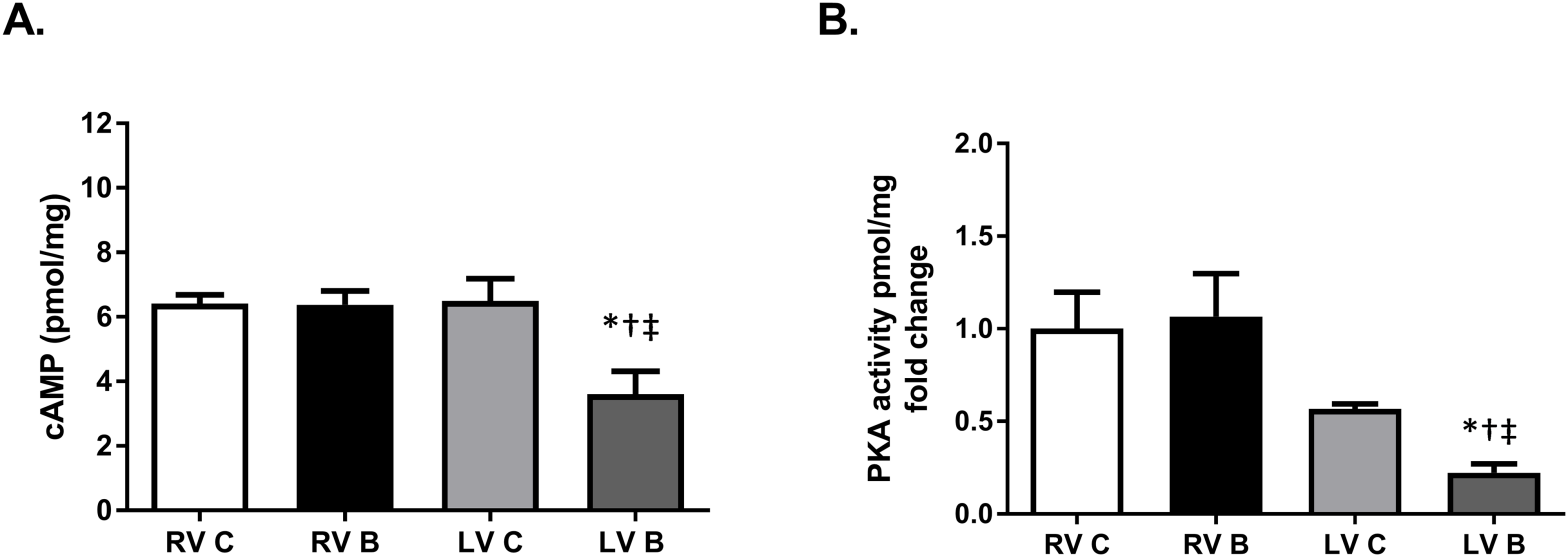
cAMP production and PKA activity post-burn. (A) cAMP production in membrane fractions isolated from right and left ventricles and (B) PKA activity in cytosolic fractions from right and left ventricles, seven days post-burn. Data are expressed as the mean ±SEM. Statistical analysis was performed using a one-way ANOVA. n = 6–9; p = 0.006 for cAMP; p = 0.004 for PKA; RV C: right ventricle control; RV B: right ventricle burned; LV C: left ventricle control; LV B: left ventricle burned; cAMP, cyclic adenosine monophosphate; PKA, protein kinase A; *, p<0.05 vs LV C; †, p<0.05 vs RV C; ‡, p<0.05 vs RV B.

Following activation of β-ARs by catecholamines, G-proteins regulate adenylyl cyclase activity. The type of G-protein released from the β-AR complex determines whether adenylyl cyclase activity is inhibited (G_αi_) or stimulated (G_αs_). Adenylyl cyclase is the primary producer of cytosolic cAMP, the activator of PKA, a kinase that phosphorylates proteins that regulate cardiac contractility, relaxation, and function. [5] Surprisingly, despite a post-burn increase in β_1_-AR and G_αs_ protein expression in the right ventricle, cAMP production and PKA activity remained unchanged. In contrast, as would be expected with increased expression of G_αi_, burn injury significantly reduced left ventricular cAMP production and PKA activity (Fig 5A and 5B; p = 0.006 and 0.004).

### Burn injury alters expression of VEGF mRNA in the left ventricle and IGF-1 mRNA in the right ventricle

**Fig 6:**
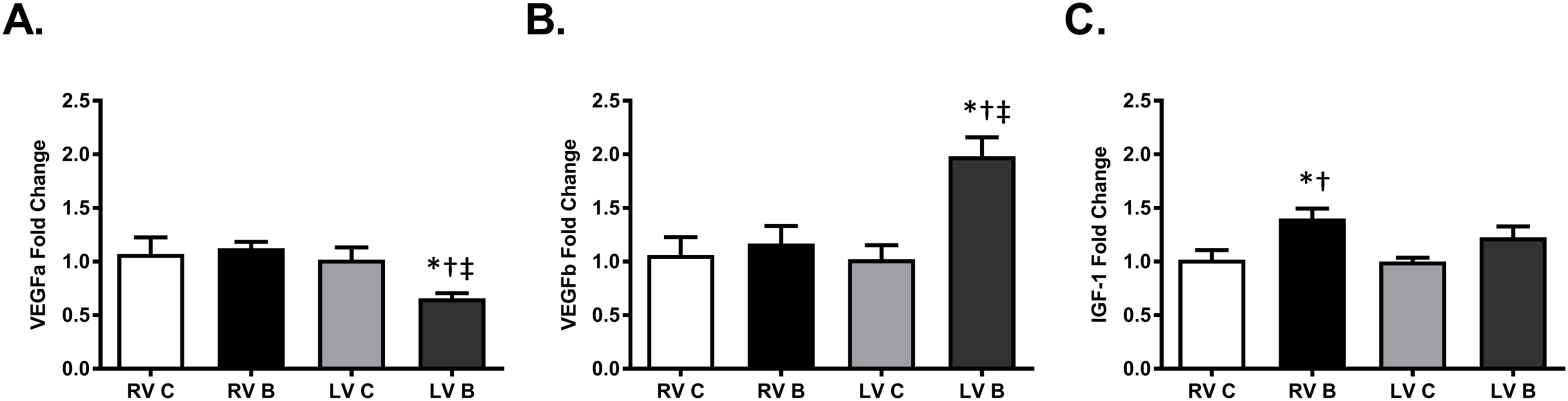
VEGF and IGF-1 gene expression post-burn. PCR measurement of VEGF-A (A), VEGF-B (B), and IGF1 (C) mRNA expression in right and left ventricles, seven days post-burn. Controls were nonburned animals. Data are expressed as the mean ± SEM. Statistical analysis was performed using a one-way ANOVA. n = 6–9; RV C: right ventricle control; RV B: right ventricle burned; LV C: left ventricle control; LV B: left ventricle burned; VEGF, vascular endothelial growth factor; IGF1, insulin-like growth factor 1; *, p<0.05 vs LV C; †, p<0.05 vs RV C; ‡, p<0.05 vs RV B.

Drake et al., showed, in two models of right ventricular failure, that vascular endothelial growth factor (VEGF) mRNA and insulin-like growth factor 1 (IGF-1) mRNA expression was significantly decreased. [20] Additionally, there is evidence that VEGF gene expression is upregulated upon β-AR stimulation [21]. In our study, VEGF-A mRNA expression was significantly decreased while expression of VEGF-B mRNA was significantly increased in the left ventricle post-burn (Fig 6A and 6B; p = 0.01; 0.002). There were no changes in expression of VEGF in the right ventricle. However, IGF-1 mRNA expression was significantly increased in the right ventricle with no statistical change observed in the left ventricle (Fig 6C; p = 0.02).

## Discussion

In disease conditions, sympathetic nervous system (SNS) tone is increased in response to either reduced cardiac output or contractility. The enhanced SNS tone causes an increased secretion of neurohormones such as norepinephrine and epinephrine. β-AR stimulation initially enhances cardiac contractility but prolonged catecholamine release and subsequent β-AR stimulation triggers a series of adaptive signaling cascades that will eventually produce cardiac dysfunction and, in some cases, heart failure. To date, there are very few mechanistic reports concerning the effect of prolonged catecholamine release in the right ventricle although there have been reports of interventricular differences in the response to β-AR stimulation. Molina et al. have shown that the response to short-term β-AR stimulation in the canine heart is significantly more robust in the right ventricle compared to the left ventricle [22]. Environmental stress conditions, which can involve increased catecholamine release, was associated with increase expression of the catecholamine synthesis enzyme phenylethanolamine N-methyltrasnferase as well as enhanced expression of β_2_-AR mRNA in the left ventricle [23]. However, the left ventricle has been the focus of most published research regarding β-AR overstimulation and cardiac dysfunction.

In heart failure, a disease state characterized by β-AR overstimulation, changes in β-AR density, signaling partners, and the termination of β-AR signaling have been well described [15, 24–27]. Sun et al. reported that total β-ARs (both β_1_- and β_2_-AR) were increased in the right ventricle of patients suffering from tetralogy of Fallot. This was accompanied by increased adenylyl cyclase activity which the authors attributed to β-ARs coupling with G_αs_ [28]. However, in contrast with the data reported by Sun et al., we did not see a concomitant increase in either cAMP or PKA in the right ventricle. While there are clear differences between our disease model and that reported by Sun et al., we hypothesize that our lack of change in cAMP levels could be due to the activation of other signaling pathways that either modulate cAMP levels or change the subcellular location of adenylyl cyclase.

β_2_-ARs can couple with both inhibitory and stimulatory G-proteins and, in the presence of chronic stimulation, it has been shown that β_2_-ARs begin to preferentially couple with G_αi_ [15, 24, 29]. This may be due to increased G_αi_ expression after prolonged catecholamine release [30]. G_αi_ inhibits adenylyl cyclase to reduce cAMP production and thus PKA activity. Similar to what has been shown in other disease conditions characterized by chronic stimulation of β-ARs, we observed both increased G_αi_ expression and reduced cAMP production and PKA activity in the left ventricle after burn injury [15, 31]. The effect of prolonged increases in catecholamine release on β_3_-ARs is not as clearly defined as the other β-ARs. Some have reported increased β_3_-AR expression after chronic stimulation whereas others and the present study were unable to determine any differences [19, 32].

Increased GRK2 and β-arrestin gene expression and/or protein expression has been associated with β-AR desensitization and downregulation after chronic β-AR stimulation [15, 24, 25]. Accordingly, we observed increased GRK2 and β-arrestin mRNA expression in the left ventricle after burn injury. This finding corroborates earlier reports of decreased β-AR affinity and the inability of exogenous β-AR stimulation to augment calcium release in burned rat cardiomyocytes [12, 33].

A previous study also reported decreased cAMP content as well as PKA activity for up to two weeks after burn injury [12]. These changes in activity were not associated with changes in the levels of PKA [12]. PKA, after activation by the binding of cAMP, phosphorylates numerous proteins underlying the mechanisms controlling cardiac lusitropy, chronotropy, and inotropy. Alterations in PKA activity can result in either hypo-phosphorylation or hyper-phosphorylation, depending upon the substrate, and can have profound effects on cardiac function [13, 34, 35]. Our finding of reduced cAMP production and PKA activity provide further evidence for the supposition that elevated catecholamine levels after burn injury leads to β-AR desensitization.

VEGF mRNA expression can be increased in a β-AR/cAMP/PKA dependent mechanism [21]. Both VEGF-A and VEGF-B regulate angiogenesis and are key components of the wound healing process. The two VEGF variants are usually co-expressed and can be found as heterodimers [36]. VEGF signaling is important for normal cardiac signaling as evidenced by the lethality of VEGF-A knockdown. Conversely, VEGF-B knockout mice are viable but have reduced cardiac size and face difficulty recovering from cardiac insults [37, 38]. Additionally, vascular diseases such as heart failure have been associated with an increase in VEGF levels [39]. We observed a decrease in left ventricular VEGF-A mRNA expression with a concurrent increase in VEGF-B mRNA expression post-burn. Increased circulating VEGF has been shown to occur in human burn patients, remains elevated until wound closure, and is associated with edema [40]. In the myocardium, increased expression of VEGF-B is associated with left ventricular cardiac hypertrophy and has been shown to occur in response to neurohormone administration. [41]. At the time-point measured in this study, we did not observe hypertrophy. However, future long-term studies will investigate whether the changes we have observed at seven days post-injury will lead to cardiac hypertrophy.

Drake et al. showed that IGF-1 gene expression was significantly increased in right ventricular hypertrophy but not in right ventricular failure [20]. As its name suggests, IGF-1 promotes growth and may contribute to the development of both physiological and pathological cardiac hypertrophy. Additionally, IGF-1 expression is increased in the presence of cardiac disease and dysfunction [42, 43]. Thus, the increase in IGF-1 may be the body’s attempt to compensate for alterations in cardiac function and morphology. We also report an increase in right ventricular IGF-1 expression following burn injury. Burn injury may promote pro-hypertrophic signaling in the right ventricle but additional studies are needed to confirm.

Changes in β-AR density, cAMP production, and GRK2 expression occurs in both right and left heart failure. However, the extent of signaling changes in the right ventricle appears to depend upon the cause of failure [44]. Therefore, the increased catecholamine release after burn injury may not be sufficient alone to produce alterations in β-AR signaling that would result in right ventricular failure. Indeed, it is often hypothesized that volume overload during resuscitation is the primary cause of right ventricular failure in burned pediatric patients [45].

## Conclusions

While our study did not observe any clear indications of right ventricular or left ventricular failure after burn injury, our data does indicate that burn injury affects the β-AR signaling pathway in the RV independently of the LV. While the LV displayed characteristic perturbations of canonical β-AR signaling expected with enhanced catecholamine release after burn injury, these data indicated non-canonical β-AR signaling may be occurring in the RV as cAMP production and PKA activity were unchanged. Our future directions are to investigate these biventricular differences over a time-course ranging from 24 hours post-injury up to 28 days post-injury.
